# Pseudocowpox virus infection in an American bison (*Bison bison*)

**DOI:** 10.1186/s12917-020-02464-7

**Published:** 2020-07-13

**Authors:** Vinay Shivanna, A. Giselle Cino-Ozuna, Cody Heskett, Douglas G. Marthaler, Charan Ganta

**Affiliations:** Kansas State University Veterinary Diagnostic Laboratory, Department of Diagnostic Medicine/Pathobiology, College of Veterinary Medicine, Kansas State University, Manhattan, Kansas 66506 USA

**Keywords:** Pseudocowpox virus, Bison, Zoonotic, Electron microscopy, Inclusions, Deep-sequencing

## Abstract

**Background:**

The present report describes a case of pseudocowpox virus (PCPV) infection in a seven-year-old female bison euthanized due to a history of declining condition and sores on the vulva and udder.

**Case presentation:**

External examination revealed multifocal, raised, keratinized plaques (0.5–2 cm) covering the skin of the ventral surface of the tail, perineum, caudoventral abdomen, udder, both inguinal recesses, and the medial aspects of both thighs. No significant gross lesions were present in the reminder of the tissues examined. Histopathological examination of the affected skin showed moderate epidermal hyperplasia with rete pegs, marked parakeratotic hyperkeratosis with crusts of degenerate neutrophils and cell debris, and few epithelial cells undergoing ballooning degeneration with occasional eosinophilic intracytoplasmic inclusion bodies (3–5 μm Bollinger body). Negative staining electron microscopy from skin revealed typical *Parapoxvirus* (PPV) particles, which were also confirmed by real-time PCR (Ct =18.6). Metagenomic analysis of the skin samples revealed only poxviruses. The bison parapox *B2L* envelope gene clustered with other parapox sequences identified from ruminants.

**Conclusions:**

This is the first report of PCPV virus infection in an American bison. Identification of novel susceptible hosts of parapox viruses sheds light on the viral evolution and highlights the importance of potential economic impact of this disease to the bison industry.

## Background

The genus *Parapoxvirus* (PPV) within the family *Poxviridae* is composed of four viruses: bovine popular stomatitis virus, orf virus, pseudocoxpox virus (PCPV) and PPV of red deer in New Zealand [1]. PPVs are ovoid (250–300 nm long and 160–190 nm in diameter) with thread-like surface tubules arranged in crisscross fashion. Their genome is composed of linear double stranded DNA of 130 kbp. PCPV infections were reported from most parts of the world and most commonly in dairy cows [2]. The common disease presentation of PPV infections includes progression through macules, papules, pustules and formation of scabs [3]. Lesions are commonly seen on muzzle of calves and teats of affected cows [4, 5]. In uncomplicated infections, scabs usually heal in a few days.

PPVs are of zoonotic importance and cause cutaneous lesions in humans from direct contact with infected animals commonly cows and sheep [6].

PPVs are epitheliotropic viruses with varying severity of infections been reported in several ungulate species such as red deer [7], reindeer [8], chamois, ibex [9], musk oxen [10], camels [11], gazelles [12] and wild Japanese serows [13]. Infections have also been reported in non-ungulates such as red squirrels, gray squirrels [14], seals [15] and pygmy chimpanzee [16]. Occurrence of PPV infections in new species can cause severe infections and pose a significant threat to the entire population of the new species [14] . PPV in general and PCPV infections in particular have not been reported previously in the American bison. We report and describe the first case of PCPV in bison, potential pathogenesis of PCPV in bison and highlight potential economic impact of this disease in the bison industry.

## Case presentation

### Case history, gross and histopathological findings

**Fig. 1:**
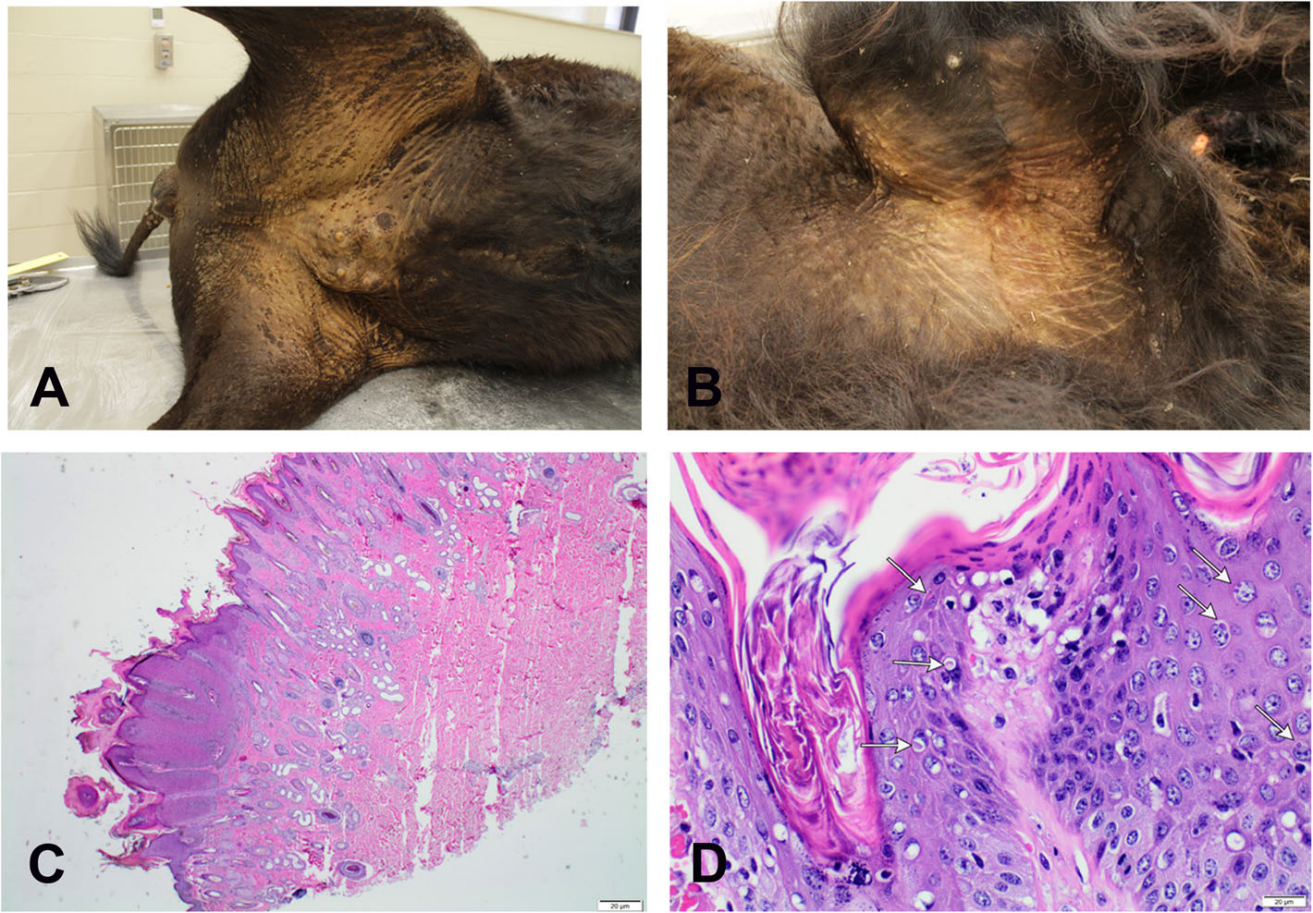
Gross and histopathological lesions of PPV infection in American bison. **a**, **b** Multiple raised keratinized nodules on the skin of ventral aspect of tail, perineum, caudoventral abdomen, udder, inguinal recesses, medial aspect of thighs and axilla. **c**, **d** Histopathological examination of the skin nodules. There is marked epidermal hyperplasia with parakeratotic hyperkeratosis and ballooning degeneration of keratinocytes within the stratum corneum and stratum spinosum. Multifocally, swollen keratinocytes contain eosinophilic intracytoplasmic inclusion bodies (white arrows)

A seven-year-old female intact American bison from the Konza Prairie Biological Station (KPBS) was presented for necropsy examination at Kansas State Veterinary Diagnostic Laboratory (KSVDL). The bison was humanely euthanized by KPBS following a clinical history of poor and declining condition prior to submission to KSVDL. Humane euthanasia was performed by KPBS using the gunshot method according to the American Veterinary Medical Association (AVMA) guidelines. During the euthanasia, multiple cutaneous nodules were noticed in the vulva and udder, and a mucus discharge was seen from the nose. A complete postmortem examination revealed lesions limited to the skin. There were multiple wart-like proliferative nodules on the ventral tail, perineum, caudoventral abdomen, udder, both inguinal recesses, and the medial aspects of both thighs, and axillary regions (Fig. 1a and b). Skin nodules were keratinized, raised, proliferative, 0.5–2 cm in diameter and became gradually fewer and smaller in size (0.2–0.5 cm) distal to the thighs. Smaller nodules (0.2–0.5 cm) also covered both axillae and the hind limbs distal to the thigh. No significant gross lesions were observed in the remaining of the organs examined.

Multiple skin sections were fixed in 10% neutral buffered formalin immediately after necropsy, and routinely processed for histopathologic examination in an automated tissue processor and embedded in paraffin. Slide-mounted tissue sections (~ 4 um thick) were stained with hematoxylin and eosin and evaluated by a certified veterinary anatomic pathologist. Histopathology of multiple skin nodules (Fig. 1c, d) consistently showed moderate epidermal hyperplasia, swollen keratinocytes with elongated rete pegs and parakeratotic hyperkeratosis that contain focal aggregates of degenerate neutrophils. Multifocally, within the epidermis, the hyperplastic stratum spinosum and stratum corneum had undergone ballooning degeneration with occasional eosinophilic intracytoplasmic inclusions measuring 5–15 μm diameter (white arrows) and pyknotic nuclei (Fig. 1c, d).

### Detection of PPV by real-time PCR and electron microscopy

Generic PPV real-time PCR testing occurred at Texas Veterinary Medical Diagnostic Laboratory (TVMDL). A representative section of the skin nodule submitted to Texas Veterinary Medical Diagnostic Laboratory (TVMDL) was positive for PPV by real-time PCR with a Ct value of 18.6, confirming strong positivity for PPV.

**Fig. 2:**
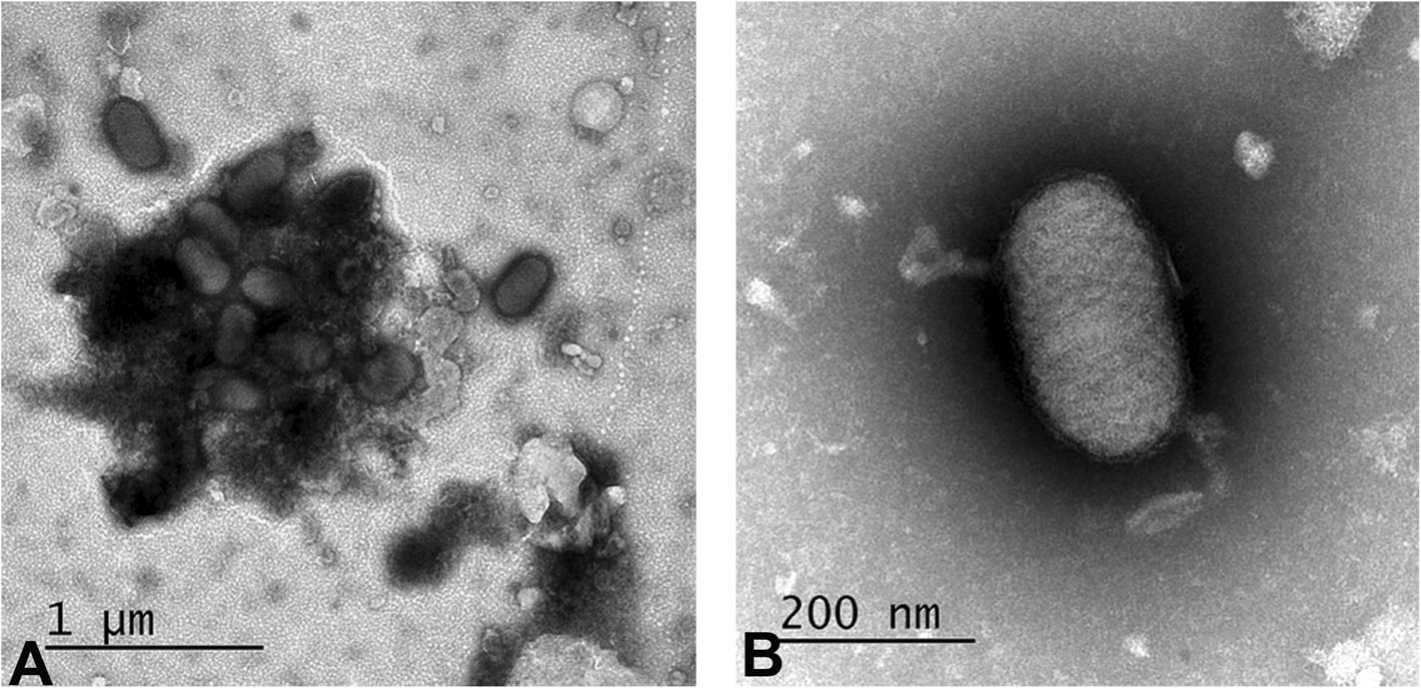
Electron microscopic examination of the suspension obtained from the skin nodules. Negatively stained aggregates of parapox viral particles (**a**) and individual PPV particle revealing ovoid virions measuring approximately 125 nm by 285 nm with spirally arranged surface proteins

Skin nodules were processed for visualizing viral particles by negative staining. The suspensions obtained from ground skin nodules were mixed with 2% phosphotungstic acid and nebulized onto EM grids. The negatively-stained grids examined by electron microscopy revealed characteristic parapox viral particles that were oval in shape (approx. 260 nm long and 160 nm wide) with a spirally organized surface tubules (Fig. 2).

### Deep sequencing

**Fig. 3:**
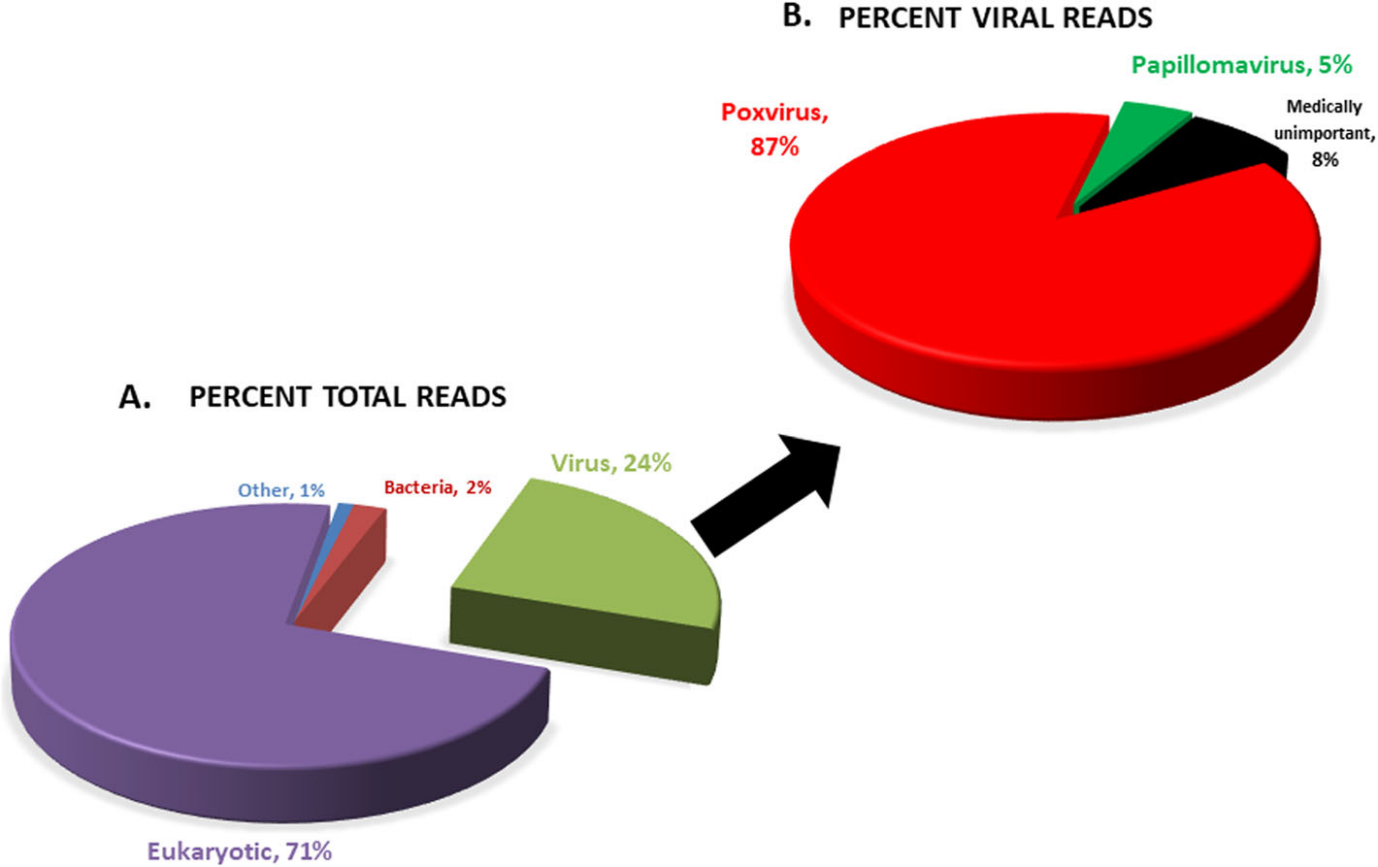
Metagenomic sequencing analysis of skin samples from American bison infected with PPV. **a** Pie chart showing 24% percentage of total reads belonging to viruses. **b** Pie chart showing poxviruses constituting 87% and papillomaviruses constituting 5% of the total virus reads

Sample processing and metagenomic sequencing was performed similar to previously described methods [17, 18]. Skin samples with lesions were processed using the Illumina Nextera XT Library Preparation Kit, according to manufacturer’s instructions. Resulting libraries were analyzed using the Agilent 2100 Bioanalyzer and Qubit 2.0 (Thermofisher). Libraries were pooled in equimolar quantities and sequenced on an Illumina MiSeq using 250 bp paired end reads. Metagenomic deep sequencing of skin samples with the lesions revealed 71% of the reads to be host specific, 24% of the reads were specific to viruses, 2% were associated with non-pathogenic bacteria, and 1% of the reads were medically unrelated. Of the virus specific reads, 87% of the reads were identified as poxvirus, 5% of the reads were identified as *Papillomaviridae,* and the remaining of the reads were not of veterinary significance. The 5% reads that matched *Papillomaviridae* were isolated, and further analysis showed that these reads were conserved between the pox - and papillomaviruses. Multiple skin samples also tested negative for papillomavirus by immunohistochemistry (data not shown). Of the poxvirus related reads, 96% of the reads were identified as PCPV and 2% were identified as orf virus (Fig. 3).

### Phylogenetic analysis

**Fig. 4:**
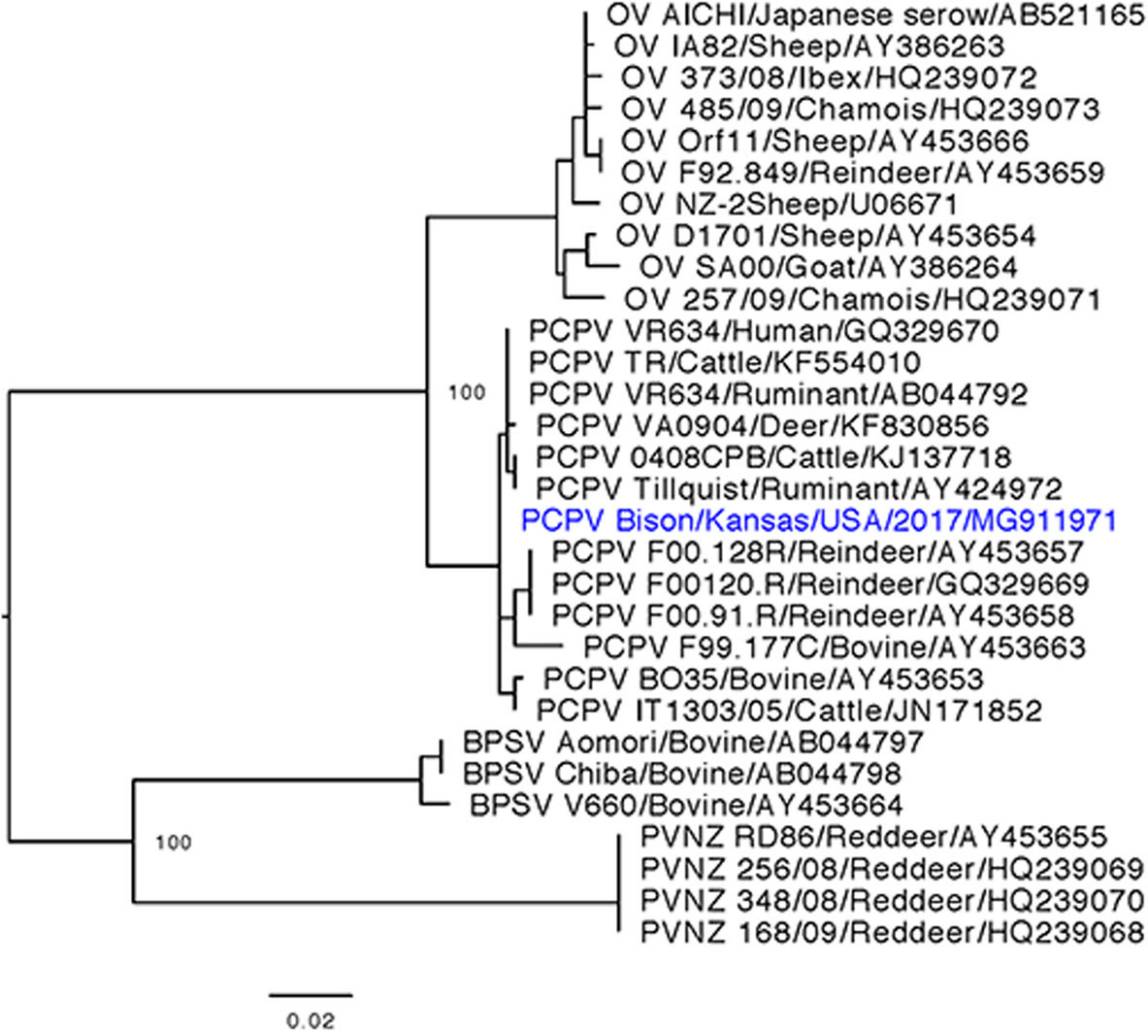
Phylogenetic tree generated from alignment of partial B2L gene sequence using MUSCLE and Maximum likelihood method with 500 replicates implemented in PHyML. The Bison related PPV clustered with other PCPV viruses and was found to be closely related to bovine PCPV

*B2L* gene amplification, sequencing and phylogenetic analysis has been used previously for identification and classification of unknown PPVs [7, 8, 19]. To further classify the PPV, the 594 bp part of the *B2L* gene was amplified from the skin nodules and Sanger sequenced (Genewiz) using previously described primers and methods [8, 9]. Phylogenetic analysis of the *B2L* gene included representative sequences previously described [9] and our newly sequenced strain. Sequences were aligned using MUSCLE [20], Maximum likelihood phylogenetic tree with 500 bootstrap replicates was created using PHyML [21]. Phylogenetic analysis illustrated that the bison PPV was closely related to bovine PCPV (Fig. 4).

## Discussion and conclusions

The present study reports the first case of PCPV virus infection in American bison (*Bison bison*) that was euthanized for declining condition and sores on the vulva and udder. Gross necropsy and histopathological examination confirmed the presence of pox viral infection. Further, real-time PCR and electron microscopy characterized the causative agent as a PPV. Phylogenetic analysis based on the *B2L* gene confirmed the causative agent as PCPV. The presence of PCPV and absence of other infectious agents in skin lesions was confirmed by metagenomic sequencing.

PCPV infections are generally reported as mild infections with only a few animals affected in a herd, and commonly seen in milking cows as skin infections of teats, udder and foot. However, an unusual presentation of pseudocowpox associated outbreak with pustular ulcerative vulvovaginits was reported in a Swedish dairy herd of approximately 80 cows. Among the affected cows, 90% of the cows had vesicles, papules and scabs affecting the vulval and vaginal mucosa [22]. An outbreak of PCPV affecting 14 of 17 male cattle was also reported from southern Brazil. Histological examination of affected tissues showed acanthosis with parakeratotic hyperkeratosis, ballooning degeneration of superficial keratinocytes and thickening of corneal layer. Electron microscopic examination of scab samples showed presence of oval shaped (260 nm X 160 nm) enveloped particles. The histological features and electron microscopic appearance of viral particles seen in the present study were consistent with these reported features of unusual presentation of PCPV infection [23]. Further, phylogenetic analysis based on the *B2L* gene was used to identify and classify the causative agent as a PCPV [23]. Similar to this report, in the present study, phylogenetic analysis based on the *B2L* gene showed that the bison PPV is closely related to bovine PCPV virus. Phylogenetic analysis showed clear segregation of different viruses into their respective genus groups with all PCPV clustering together. The bison PCPV was found to cluster closely (100%) with other PCPV of cattle origin.

Metagenomic sequence analysis revealed a majority of the reads were related to viruses, of which 87% corresponded to poxviruses and 5% of the viral reads were papillomavirus related. The small percentage of reads matching papillomavirus were found to be the conserved gene sequences that are common to poxviruses and papillomaviruses [24].

Cases of severe atypical PPV infections were previously reported in sheep from the United States [25]. These atypical cases were present in sheep over 12 months of age, characterized by lesions similar to classic orf but more severe, did not resolve spontaneously, and led to debilitating conditions in all affected sheep [25]. Diagnosis of PPV was made based on histological and electron microscopic examination but lacked molecular characterization. Histopathologically, all PPV infections have similar lesions hence are indistinguishable.

Investigating the PPV outbreaks in Finnish reindeer using phylogenetic analysis based on the *B2L* gene, showed that the PPV outbreak of 1999–2000 was closely related to bovine PCPV whereas the PPV outbreak of 1992–93 was closely related to sheep orf virus [8]. PPV outbreaks were reported in red deer from New Zealand in 1986 [7] and from Italy in 2008–09 [26]. Phylogenetic analysis based on the *B2L* gene of the PPV from red deer in Italy showed that the virus was closely related to New Zealand PPV of red deer [9]. Further genetic analysis of the bison PPV is required to determine if it is a bovine PCPV that accidently infected bison or is genetically distant enough to be classified as bison variant of PCPV virus.

Transmission of poxvirus to a new species can have detrimental effects on the new population. When squirrel pox virus was transmitted from grey squirrels to red squirrels in Great Britain, it led to significant losses in the red squirrel population [14].

Although the bison PPV from the current study is closely related to bovine PCPV, the pathological lesions seen in the bison were very distinct from PCPV lesions seen in cattle. The source of the PPV infection in bison is not known. PCPV virus infection in cattle is generally a mild infection characterized by small “ring” or “horseshoe”- shaped papules on the teats and udder that progress to form vesicles or pustules and heal by forming scabs [2]. In comparison to cattle, the lesions seen in bison were more severe with the papules extending over a larger area of the body, caused ill thrift and significantly affected productivity. Several primary infections could predispose or increase the severity of PPV infection. No histologic lesions suggestive of any immunosuppressive viral or bacterial infections were seen in various other tissues examined.

In conclusion, the present report describes an atypical and severe form of PCPV infection in a new host species. The infection was characterized by multifocal raised nodules, extending over large cutaneous areas in an American bison. Definitive identification of the PCPV was made by gross and histopathological examination, real-time PCR, electron microscopy, metagenomic sequencing and phylogenetic analysis based on the *B2L* gene. Further investigation to determine the pathogenicity and zoonotic potential of this bison PCPV is warranted.

## Data Availability

The datasets generated and/or analyzed during the current study are available in the NCBI, Nucleotide repository, [https://www.ncbi.nlm.nih.gov/nuccore/MG911971]. All data presented here is available upon request to corresponding author.

## Abbreviations

PCPV: Pseudocowpox virus
PPV: Parapox virus
PVNZ: Parapoxvirus of New Zealand
PCR: Polymerase chain reaction
DNA: Deoxyribonucleic acid
BVDV: Bovine viral diarrhea virus
EM: Electron Microscopy
KSVDL: Kansas State Veterinary Diagnostic Laboratory
TVMDL: Texas A&M Veterinary Medical Diagnostic Laboratory
KPBS: Konza Prairie Biological Station
AVMA: American Veterinary Medical Association
Ct: Cycle threshold
